# *Notes from the Field:* First Case in the United States of *Neisseria gonorrhoeae* Harboring Emerging Mosaic *penA*60 Allele, Conferring Reduced Susceptibility to Cefixime and Ceftriaxone

**DOI:** 10.15585/mmwr.mm6949a5

**Published:** 2020-12-11

**Authors:** Michael A. Picker, Ronald J. Knoblock, Holly Hansen, Ilene Bautista, Rey Griego, Lora Barber, William Bendik, Kit Lam, Elizabeth Adelman, Zuwen Qiu-Shultz, Brian H. Raphael, Cau D. Pham, Ellen N. Kersh, Hillard Weinstock, Sancta B. St. Cyr

**Affiliations:** ^1^Southern Nevada Public Health Laboratory of the Southern Nevada Health District, Las Vegas; ^2^University Medical Center of Southern Nevada, Las Vegas; ^3^Office of Epidemiology and Disease Surveillance, Southern Nevada Health District, Las Vegas; ^4^Division of STD Prevention, National Center for HIV/AIDS, Viral Hepatitis, STD, and TB Prevention, CDC.

In November 2019, the Southern Nevada Public Health Laboratory of the Southern Nevada Health District (SNHD) identified a male urethral gonococcal isolate later demonstrating reduced susceptibility to cefixime (minimum inhibitory concentration [MIC] = 2.0 *μ*g/mL) and ceftriaxone (MIC = 1.0 *μ*g/mL) but susceptible to azithromycin (MIC = 0.25 *μ*g/mL). Molecular testing by CDC in the United States revealed the emerging mosaic *penA*60 allele, first identified in Japan in 2016 (*1*), which confers reduced susceptibility to cephalosporins and increases the risk for treatment failure. The *penA*60 allele has been identified in China (*2*), Canada (*3*,*4*), Denmark (*5*), Australia (*6*), France (*7*), and the United Kingdom (*8*). The Nevada case is the first identified case of a *Neisseria gonorrhoeae* isolate harboring the mosaic *penA*60 allele reported in the United States.

The SNHD in Las Vegas, Nevada, participates in the Gonococcal Isolate Surveillance Project (GISP), the U.S. sentinel surveillance program for monitoring antibiotic-resistant gonorrhea (https://www.cdc.gov/std/gisp/default.htm). As part of the project, the Sexual Health Clinic at SNHD collects urethral samples for gonococcal culturing from the first 25 men each month, on average, with symptoms consistent with urethral gonococcal infection. All *N. gonorrhoeae* isolates are sent to the Texas Department of State Health Services Laboratory, part of CDC’s Antibiotic Resistance Laboratory Network, where MICs are determined via agar dilution. Susceptibility results are then reported back to SNHD and CDC.

This SNHD gonococcal isolate was collected from an HIV-negative, heterosexual man with penile discharge and dysuria in October 2019; he was empirically treated with the recommended regimen of ceftriaxone (250 mg) given intramuscularly plus azithromycin (1 g) administered orally (*9*). Nucleic acid amplification tests (NAATs) and cultures collected from the patient’s rectum and pharynx were negative for *N. gonorrhoeae*. After the susceptibility results from the urethral isolate were confirmed by CDC in late November 2019, he was encouraged to return to SNHD for repeat testing and evaluation.

At his follow-up visit at the end of November 2019, the patient reported that his urethral symptoms had resolved. Repeat gonococcal tests at all anatomic sites returned negative results. During this visit, he reported three female sexual partners (one main partner and two casual partners) during the 2 months before he sought care in October 2019. SNHD was unable to contact his main partner, initially because of a 2-month visit overseas with her family beginning in early October 2019 and then because of nonresponse upon her return in January 2020. The patient relayed to SNHD that this main partner had been tested and treated for gonorrhea while in China. It was her communication to him of her diagnosis and treatment, in addition to his onset of symptoms, that prompted him to seek medical evaluation in October 2019. SNHD staff members were able to locate one of his casual partners in December 2019. This casual partner visited SNHD for testing and treatment and received ceftriaxone (250 mg) intramuscularly and azithromycin (1 g) orally. Her genital and extragenital NAATs and subsequent cultures were negative for *N. gonorrhoeae*. His other casual partner was anonymous and could not be contacted because locating information was unavailable.

To identify other cases of *N. gonorrhoeae* with the *penA*60 allele in the southern Nevada area, in December 2019, SNHD provided CDC with all *N. gonorrhoeae* NAAT-positive specimens from all SNHD clinics (257 remnant NAATs collected during September 2019–November 2019). Culture-independent molecular testing for the mosaic *penA*60 allele of the remnant NAATs (*6*) identified no additional isolates with the mosaic *penA*60 allele. Approximately 5,500 gonococcal isolates were submitted for testing nationwide as part of GISP during January 2019–December 2019. No other isolates had MICs as high as those for the isolate in the Nevada case (2.0 *μ*g/mL for cefixime and 1.0 *μ*g/mL for ceftriaxone). An advisory was sent to state and local jurisdictions in early 2020; the investigation was stopped in February 2020 because no other isolates of concern had been identified.

This *N. gonorrhoeae* isolate with the mosaic *penA*60 allele and reduced susceptibility to cefixime and ceftriaxone is the first of its kind detected in the United States. The results of this investigation supported local acquisition of the infection, but the origin of the isolate remains unclear. Its MIC is the highest for ceftriaxone observed since GISP began monitoring ceftriaxone and other antimicrobial susceptibilities in 1986. To date, continued spread of this isolate has not been seen in southern Nevada or GISP. Despite the susceptibility patterns identified in this case, no treatment failures in the United States using the current recommended regimen of ceftriaxone and azithromycin have been reported. This case highlights the utility and importance of surveillance programs like GISP as effective tools in identifying emerging antimicrobial-resistant pathogens that can negatively impact patient outcomes and threaten public health.
